# Longevity and replenishment of human liver-resident memory T cells and mononuclear phagocytes

**DOI:** 10.1084/jem.20200050

**Published:** 2020-06-30

**Authors:** Laura J. Pallett, Alice R. Burton, Oliver E. Amin, Sergio Rodriguez-Tajes, Amit A. Patel, Nekisa Zakeri, Anna Jeffery-Smith, Leo Swadling, Nathalie M. Schmidt, Anna Baiges, Amir Gander, Dominic Yu, David Nasralla, Farid Froghi, Satheesh Iype, Brian R. Davidson, Douglas Thorburn, Simon Yona, Xavier Forns, Mala K. Maini

**Affiliations:** 1 Division of Infection and Immunity, Institute of Immunity and Transplantation, University College London, UK; 2 Liver Unit, Hospital Clinic, August Pi i Sunyer Biomedical Research Institute (IDIBAPS) and Centro de Investigación Biomédica en Red de Enfermedades Hepáticas y Digestivas (CIBEREHD), University of Barcelona, Barcelona, Spain; 3 Division of Medicine, University College London, London, UK; 4 Barts Liver Centre, Blizard Institute, Barts and The London School of Medicine and Dentistry, Queen Mary University of London, London, UK; 5 Division of Surgery, University College London, London, UK; 6 Liver Transplant Unit, Royal Free Hospital, London, UK; 7 Institute of Dental Sciences, Hebrew University, Jerusalem, Israel

## Abstract

The human liver contains specialized subsets of mononuclear phagocytes (MNPs) and T cells, but whether these have definitive features of tissue residence (long-term retention, lack of egress) and/or can be replenished from the circulation remains unclear. Here we addressed these questions using HLA-mismatched liver allografts to discriminate the liver-resident (donor) from the infiltrating (recipient) immune composition. Allografts were rapidly infiltrated by recipient leukocytes, which recapitulated the liver myeloid and lymphoid composition, and underwent partial reprogramming with acquisition of CD68/CD206 on MNPs and CD69/CD103 on T cells. The small residual pool of donor cells persisting in allografts for over a decade contained CX3CR1^hi^/CD163^hi^/CD206^hi^ Kupffer cells (KCs) and CXCR3^hi^ tissue-resident memory T cells (T_RM_). CD8^+^ T_RM_ were found in the local lymph nodes but were not detected egressing into the hepatic vein. Our findings inform organ transplantation and hepatic immunotherapy, revealing remarkably long-lived populations of KCs and T_RM_ in human liver, which can be additionally supplemented by their circulating counterparts.

## Teaser

Pallett et al. identify mononuclear phagocyte and T cell subsets able to reside in the human liver for over a decade, probing their retention and replenishment using HLA-mismatched allografts and hepatic venous sampling.

## Graphical Abstract

**Figure:**
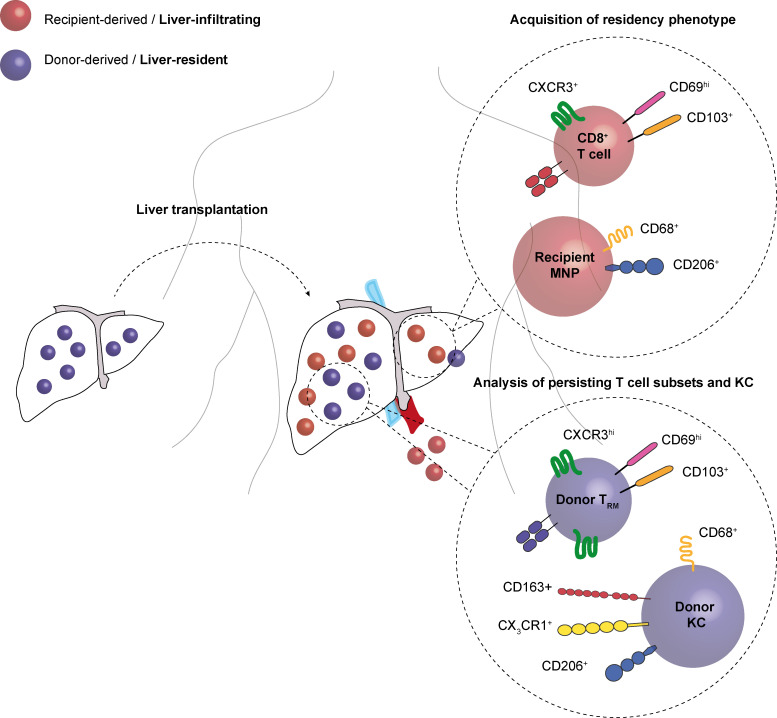

## Introduction

Tissue-specific leukocytes play vital roles in shaping the local immune landscape, mediating protective and pathogenic responses to a variety of threats. Characterization of their adaptations to specific niches will allow tailored manipulation to optimize frontline immunosurveillance while preserving organ integrity. It has long been recognized that each tissue has its own specialized population of macrophages adapted to perform unique functions, such as scavenging surfactant in the lungs and microbial products or iron in the liver (Davies et al., 2013; Ginhoux and Guilliams, 2016; Guilliams et al., 2018). More recently, a number of innate-like tissue-resident lymphocytes have also been recognized, as well as large populations of classical αβTCR memory CD4^+^ and CD8^+^ T cells compartmentalized in both lymphoid and nonlymphoid organs, that play critical roles in pathogen and tumor surveillance (Fan and Rudensky, 2016; Masopust and Soerens, 2019; Szabo et al., 2019).

The liver acts as a central hub for many systemic metabolic pathways and plays a key tolerogenic role in its position as a firewall between the portal venous blood from the gut and the systemic circulation (Protzer et al., 2012). It contains the largest population of macrophages in the body, known as Kupffer cells (KCs), which are typically identified by their characteristic intra-sinusoidal location and distinct morphology (Krenkel and Tacke, 2017). The intravascular localization of KCs and the specialized liver sinusoidal endothelium equip this organ with a unique capacity to prime T cells; KCs are able to prime effective CD8^+^ T cells, whereas priming by liver sinusoidal endothelium or hepatocytes leads to dysfunctional responses (Bénéchet et al., 2019; Limmer et al., 2000). Murine fate-mapping studies have identified hepatic KCs as fetal yolk sac–derived, sessile macrophages that are stable and long-lived in homeostatic conditions (Schulz et al., 2012; Yona et al., 2013). However, when murine liver-resident macrophages are experimentally depleted, the space in the niche can then be efficiently replenished by peripheral bone marrow–derived monocytes that acquire KC features (Klein et al., 2007; Scott et al., 2016). Signals from the liver niche that are able to impose a KC-type phenotype on incoming mononuclear phagocytes (MNP) have begun to be identified (Sakai et al., 2019). Recent single-cell RNA-sequencing profiling of human fetal and adult livers (Aizarani et al., 2019; MacParland et al., 2018; Popescu et al., 2019; Ramachandran et al., 2019) has revealed distinct clusters of macrophages, with some transcriptional overlap with the hepatic yolk sac and peripheral infiltrating subsets defined in mice. However, the local longevity and/or peripheral replenishment of hepatic macrophages in humans remain unclear.

Similarly, approaches such as intravital imaging and parabiosis have defined the capacity of a subset of murine memory CD8^+^ T cells to mediate resident hepatic immunosurveillance, patrolling the sinusoidal vasculature in a CXCR6-dependent manner (Fernandez-Ruiz et al., 2016; Tse et al., 2014). We have identified an analogous subset of CXCR6^hi^ CD69^+^CD103^+^CD8^+^ T cells in the human liver that are transcriptionally, phenotypically, and functionally distinct from memory CD8^+^ T cells in the periphery (Pallett et al., 2017). This liver-compartmentalized population has been assumed to represent bona fide CD8^+^ tissue-resident memory T (T_RM_) cells on the basis of its core signatures (Kumar et al., 2017; Pallett et al., 2017), but it has not been possible to formally determine whether it has the capacity for long-lived tissue retention, nor whether it can be replenished from the periphery.

Thus, studies in mice have allowed considerable insights into the tissue residence, longevity, and replenishment of myeloid and lymphoid cell types in different organs, including the liver. The human counterparts of liver-resident macrophages and T_RM_ cells have begun to be identified and profiled. An understanding of the longevity of these frontline human liver sentinels and their potential for egress or replenishment from peripheral subsets is needed in order to harness them for immunotherapy. To address this, we took advantage of the fact that the liver is typically transplanted without human leukocyte antigen (HLA) matching, allowing donor (liver-resident) and recipient (blood-derived) leukocytes extracted from allografts months to years later to be distinguished by HLA staining and flow-cytometric analysis.

## Results and discussion

### Distinguishing the resident and infiltrating immune landscape of the liver using HLA-mismatched allografts

**Figure 1.**
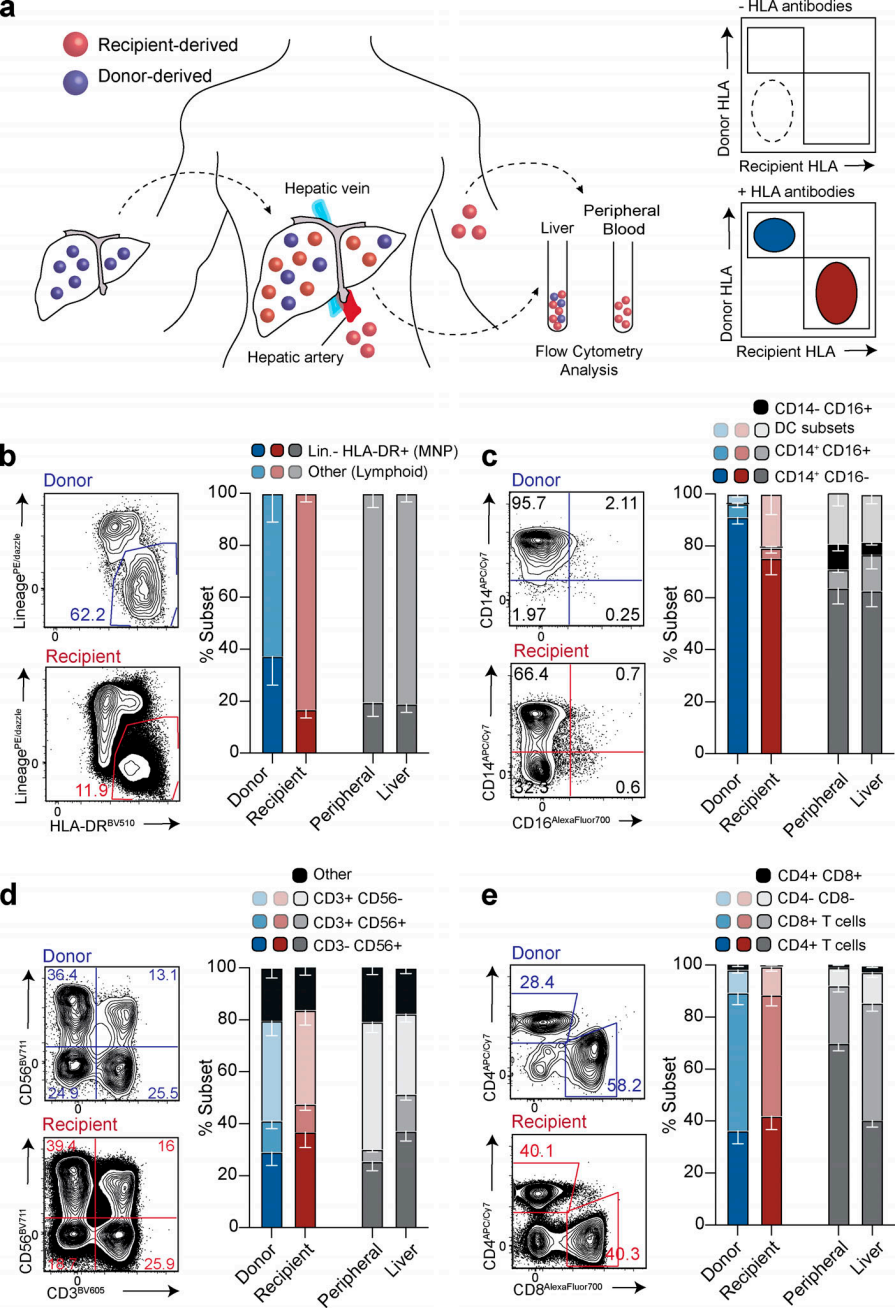
**Characterization of the persisting donor-derived and infiltrating, recipient-derived hepatic immune landscape. (a)** Schematic showing experimental design for flow-cytometric analysis: identification of donor-derived (blue) and recipient-derived (red) leukocytes (live, singlet, CD45^+^ cells) on the basis of class I HLA-haplotype mismatch, in peripheral blood and liver at time of allograft removal. **(b–e)** Representative flow-cytometric plots and summary cell frequencies of (b) lineage^−^ (CD66b^−^CD3^−^CD56^−^CD19^−^CD20^−^) HLA-DR^+^ “myeloid-lineage” cells (*n* = 5); (c) MNP subsets, including DCs, on the basis of CD14 and CD16 expression (*n* = 5; five independent experiments); (d) lymphocyte subsets, on the basis of CD56 and CD3 expression (*n* = 6; six independent experiments); and (e) CD3^+^ T cell subset distribution based on expression CD4 and CD8 (*n* = 6; six independent experiments). Cell types were distinguished within the donor (blue) and recipient (red) leukocyte pools using the gating strategy and HLA-class I antibody staining exemplified in Fig. S1, a and b, and compared with control, nontransplanted liver tissue samples obtained during surgical removal of colorectal carcinoma metastases or HCC (liver; *n* = 8–13; 8–13 independent experiments; gray) and peripheral blood (peripheral; *n* = 8–13; 8–13 independent experiments; gray). Error bars, mean ± SEM.

**Figure S1.**
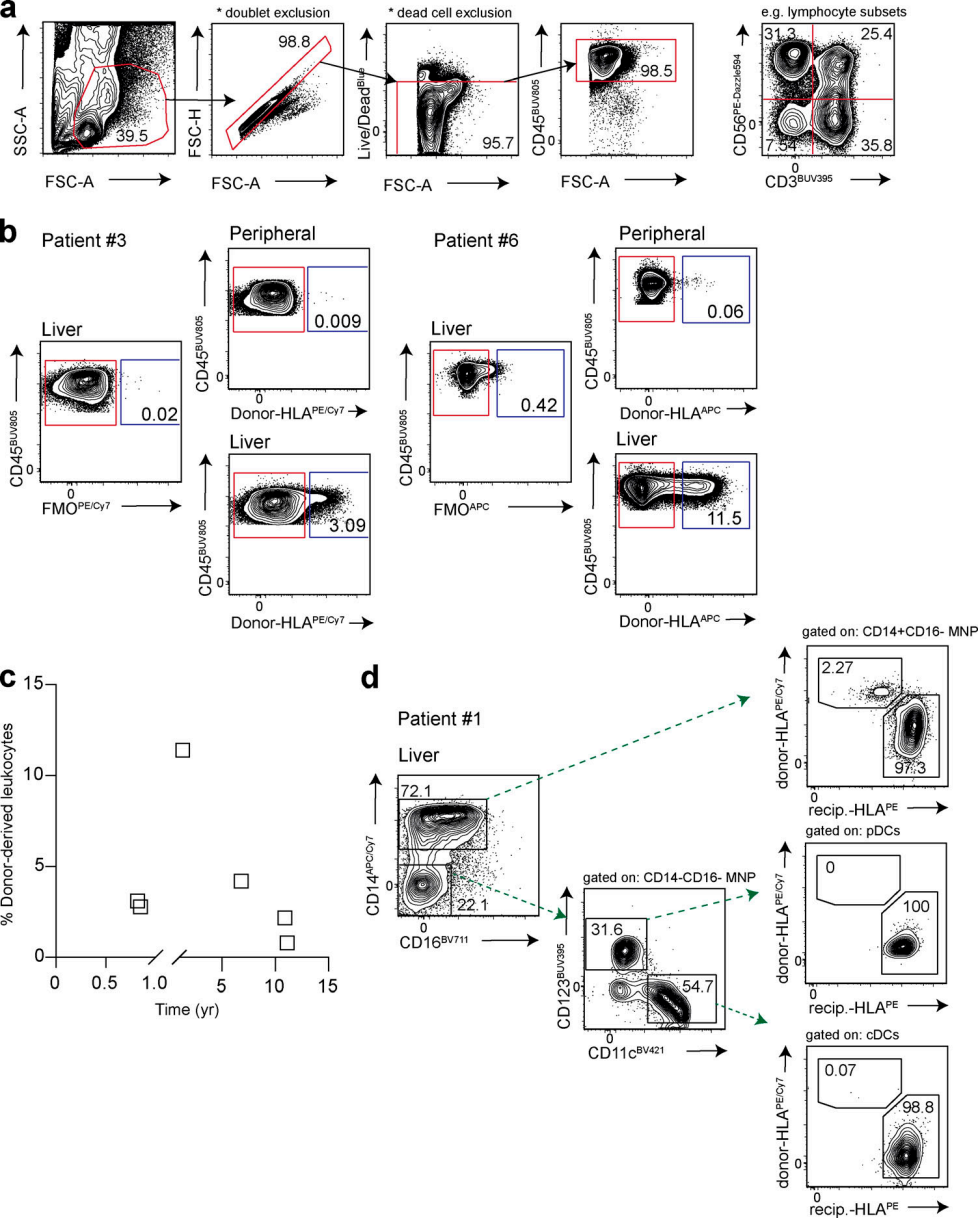
**Identification of donor-derived CD45^+^ leukocytes by flow cytometry. (a)** Representative sequential gating strategy for the identification of CD45^+^ leukocytes in human peripheral blood, liver tissue, and hepatic hilar lymph nodes (live, singlet cells) using 16-color flow cytometry. FSC-A, forward scatter; SSC-A, side scatter; Live/dead, fixable dead cell stain. **(b)** Two representative flow-cytometric plots showing identification of donor- and recipient-derived CD45^+^ leukocytes using HLA-class I monoclonal antibodies, gated using a fluorescence minus one (FMO) control. **(c)** Frequency of donor-derived CD45^+^ leukocytes (gated on live, singlet cells; *n* = 6; six independent experiments) identified in the allograft for each individual in relation to time to explant, based on monoclonal antibody staining for donor-derived HLA-class I haplotype exemplified in panel b. **(d)** Representative flow-cytometric plots showing donor and recipient HLA-class I haplotype staining exemplified in panel b on “classical” Lin^−^HLA-DR^+^CD14^+^CD16^−^ MNPs and DC subsets (Lin^−^HLA-DR^+^CD14^−^CD16^−^CD123^+^CD11c^−^ plasmacytoid DCs [pDCs], Lin^−^HLA-DR^+^CD14^−^CD16^−^CD123^−^CD11c^+^ conventional DCs [cDCs]) within an allograft.

We used HLA-specific monoclonal antibodies to distinguish donor and recipient leukocytes from liver allografts explanted months to years after transplantation into HLA-mismatched individuals (schematic, Fig. 1 a; gating strategy, Fig. S1 a; example HLA-staining, Fig S1 b). Liver allograft samples were available from between 8 mo and >11 yr after initial transplantation (at time of retransplantation for recurrent disease or complications other than chronic rejection, see Table S1 for patient characteristics). This approach allowed us to mark liver-resident progeny of known minimum longevity (using donor-HLA and time since transplant) and to investigate the influence of liver homing on blood-derived cells (recipient-HLA cells infiltrating the liver allograft). The majority of the intrahepatic immune landscape was replaced with recipient-derived leukocytes at the earliest time point examined (8 mo after transplantation, Fig. S1 c). However, a small population of donor-derived leukocytes persisted within the liver, even in the two allografts examined ∼11 yr after initial transplantation. There was no relationship between the frequency of persisting donor-origin leukocytes and the time since transplantation in this small cohort (Fig. S1 c). By contrast, there were negligible donor-derived leukocytes detectable in the blood of any of the recipients (<0.1% in all, representative stains in Fig. S1 b), arguing against systemic chimerism accounting for intrahepatic persistence. The extensive replacement of donor-derived leukocytes may have been accelerated by their rapid loss in the transplant setting, creating space in the liver niche. Depletion of donor leukocytes could be facilitated by the intravascular localization of many immune populations in the liver, allowing large numbers to be flushed out by the organ perfusion that is performed before transplantation (Pallett et al., 2017). This setting of organ transplantation with recurrent liver disease and/or allogeneic responses precluded direct extrapolation to the normal homeostatic turnover of intrahepatic populations. Instead, it provided an opportunity to assess immune cell subsets able to withstand these adverse challenges and persist long-term within the liver, and the potential for repopulation from the periphery when there is space in the niche.

We noted that within the small persisting pool of donor leukocytes, there was preferential preservation of the myeloid compartment (separated using lineage markers CD66b/CD56/CD19/CD20/CD3 and HLA-DR; Fig. 1 b). Within the donor myeloid pool, CD14^+^CD16^−^ MNP survived long-term, whereas conventional dendritic cells (DCs; CD11c^+^CD123^−^) and plasmacytoid DCs (CD11c^−^CD123^+^) were already undetectable in the earliest sampled allograft (Fig. 1 c and Fig. S1 d). However, recipient-derived myeloid cells were able to repopulate the liver with the spectrum of MNP and DC seen in control nontransplanted livers and peripheral blood (Fig. 1 c and Fig. S1 d).

By contrast, the donor lymphoid compartment contained persistent populations of T cells (CD3^+^CD56^−^), natural killer (NK) cells (CD3^−^CD56^+^) and NK-like T cells (CD3^+^CD56^+^; Fig. 1 d). Infiltrating recipient lymphocytes equilibrated to reflect the cellular composition of control nontransplanted livers, with a relative enrichment of NK and NK-like T cells compared with blood (Fig. 1 d). These data suggested that the recipient-derived fraction receives cues from the liver microenvironment to recapitulate the composition of the endogenous intrahepatic pool. To analyze this further, we compared the ratio of CD4^+^ to CD8^+^ subsets within the CD3^+^CD56^−^ T cell fraction, since a bias toward CD8^+^ T cells is a well-recognized feature of the liver (Norris et al., 1998). As expected, persisting T cells of donor origin showed a striking enrichment of CD8^+^ T cells compared with blood. Interestingly, recipient-derived T cells infiltrating the liver developed a similar enrichment of CD8^+^ over CD4^+^ T cells to that seen in healthy liver and the donor-derived fraction (Fig. 1 e). Taken together, these data pointed to inherent cues in the liver microenvironment able to tightly regulate the composition of the lymphoid pool. This contrasted with the more biased maintenance of the myeloid compartment, with selective long-lived retention of a CD14^+^CD16^−^ MNP subset.

### The human hepatic myeloid compartment contains a long-lived CX_3_CR1^hi^CD163^hi^CD14^+^ subset and can be repopulated with blood MNPs

**Figure 2.**
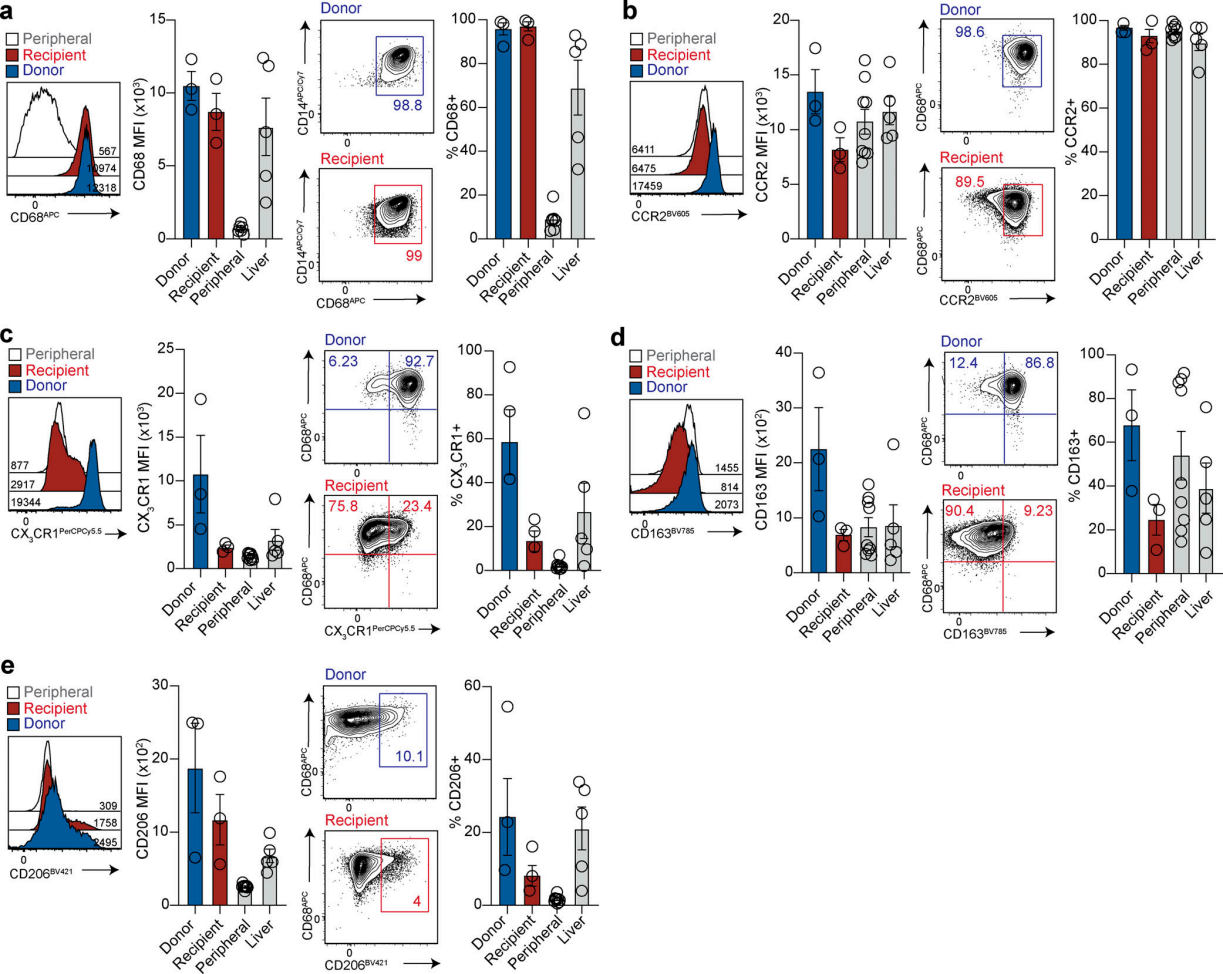
**Phenotypic comparison of long-lived donor-derived and infiltrating recipient-derived MNP.** **(a–e)** Representative flow-cytometric plots and summary data showing the expression (MFI and %) of (a) CD68 (*n* = 3; three independent experiments); (b) CCR2 (*n* = 3; three independent experiments); (c) CX_3_CR1 (*n* = 3; three independent experiments); (d) CD163 (*n* = 3; three independent experiments); and (e) CD206 (*n* = 3; three independent experiments) on donor (blue) or recipient (red) origin “classical” Lin^−^HLA-DR^+^CD14^+^CD16^−^ MNPs within allografts, compared with control, nontransplanted liver tissue samples obtained during surgical removal of colorectal carcinoma metastases or HCC (liver; *n* = 5; five independent experiments; gray) and peripheral blood (peripheral; *n* = 9; nine independent experiments; gray) “classical” Lin^−^HLA-DR^+^CD14^+^CD16^−^ MNP. Error bars, mean ± SEM.

We next aimed to better define the nature of the small population of donor-derived CD14^+^CD16^−^ MNPs that was still detectable after more than 10 yr in the liver allograft, and to ascertain whether the large fraction of peripheral, infiltrating MNPs could acquire the same phenotype. Where sufficient cells were available, we compared the phenotypic profile of the long-lived donor-derived CD14^+^CD16^−^ MNP population to their counterparts repopulating the liver from the recipient circulation, as well as those present in control nontransplant livers and blood. Donor and recipient–derived intrahepatic MNPs expressed the prototypic KC marker CD68 at uniformly high levels (by percentage and mean fluorescence intensity [MFI] expression; Fig. 2 a). CD68 was also seen in control nontransplanted liver MNPs but was minimally expressed on CD14^+^CD16^−^ MNP from the blood, implying that a population of recipient blood MNP had acquired strong expression of this prototypic marker within months of infiltrating the liver. This is in accordance with previous immunofluorescence staining of CD68 on recipient-derived intra-sinusoidal cells in human liver transplants (Ng et al., 2003) and with the recent demonstration of reprogramming of infiltrating MNPs within the murine liver (Ng et al., 2003; Scott et al., 2016; Sakai et al., 2019). Thus, CD68 represents a marker acquired by MNP upon entry into the human liver, but not one that can be used to distinguish resident embryonic progenitor-derived macrophages from infiltrating bone marrow–derived MNPs.

We therefore investigated differential expression of other flow-cytometric markers between donor- and recipient-derived CD14^+^CD16^−^ MNPs. The chemokine receptor CCR2 drives liver homing of scar-associated MNPs (Krenkel et al., 2018; Ramachandran et al., 2019) and has been recently shown to differentiate functionally distinct subsets of macrophages within the transplanted human heart, with the CCR2^−^ fraction representing the bona fide tissue-resident subset and the CCR2^+^ a proinflammatory infiltrating MNP (Bajpai et al., 2018). However, CCR2 was not discriminatory in the transplanted liver, with uniform expression found on blood, nontransplant livers, recipient- and donor-derived MNPs within the allografts, and a tendency for highest expression on the latter (Fig. 2 b). By contrast, the fractalkine receptor CX_3_CR1, required for the embryonic seeding of murine tissue-resident macrophages including KCs (Mass et al., 2016; Yona et al., 2013), was clearly enriched on long-lived donor-derived human MNPs, compared with the low expression observed on recipient infiltrating counterparts or blood MNP (Fig. 2 c).

**Figure S2.**
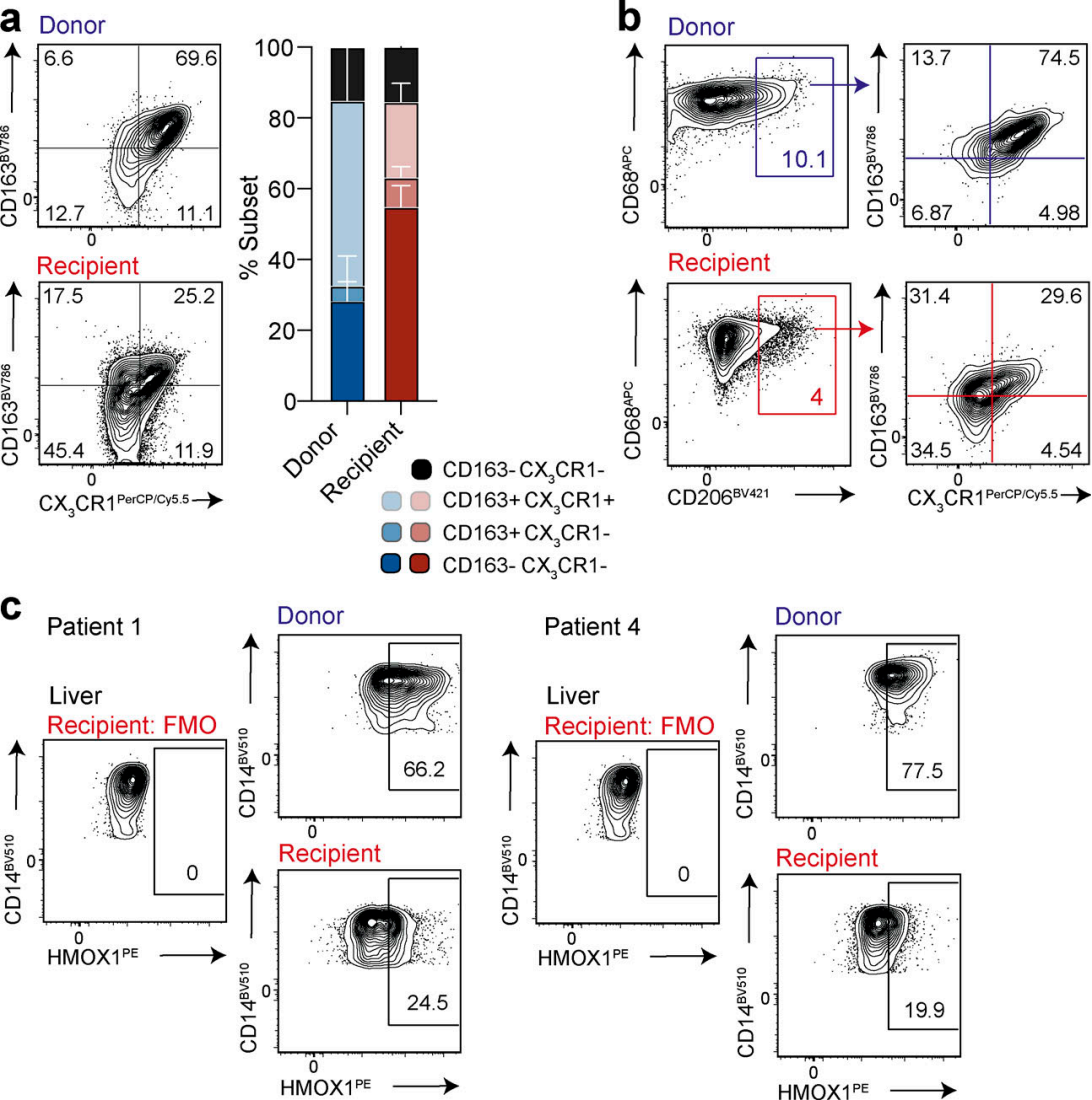
**Co-expression profiling of long-lived donor-derived and infiltrating recipient-derived MNP. (a)** Representative flow-cytometric plots and summary data showing coexpression of CD163 and CX_3_CR1 on donor (blue) or recipient (red) origin “classical” Lin^−^HLA-DR^+^CD68^+^CD14^+^CD16^−^ MNPs within allografts (*n* = 3; three independent experiments). **(b)** Representative flow-cytometric plots showing CD206, CX_3_CR1, and CD163 coexpression on CD68^+^ donor and recipient origin MNPs. **(c)** Representative flow-cytometric plots showing expression of HMOX1 on donor (blue) or recipient (red) origin “classical” Lin^−^HLA-DR^+^CD14^+^CD16^−^ MNP within allografts (*n* = 2; one independent experiment). Error bars, mean ± SEM.

By examining transcripts distinguishing tolerogenic and proinflammatory CD68^+^ subsets in overlaid gene lists of recent human liver MNP single-cell RNA profiles (Aizarani et al., 2019; MacParland et al., 2018; Ramachandran et al., 2019), we selected the mannose receptor CD206 and the scavenger receptor CD163 as candidates for further flow-cytometric analysis. Of 30 genes shared between these lists, CD163 was among the 22 genes also identified in the macrophage cluster of human fetal liver transcripts (Popescu et al., 2019), further pointing to its potential utility in marking embryonic KCs. Consistent with this, donor-derived MNPs tended to express more CD206 and CD163 (frequently coexpressed with CX_3_CR1) than their recipient-derived liver-infiltrating counterparts (Fig. 2, d and e; and Fig. S2, a and b). Preliminary data also revealed preferential expression of HMOX1 by donor compared with recipient-derived MNPs (Fig. S2 c), consistent with the role of KCs in iron metabolism (Scott and Guilliams, 2018; Scott et al., 2016); future studies could include additional emerging hepatic macrophage markers such as Timd4, MARCO, VISG4, and CD207 (Aizarani et al., 2019; MacParland et al., 2018; Mass et al., 2016; Popescu et al., 2019; Ramachandran et al., 2019; Sierro et al., 2017). Taken together, these data show for the first time that CD68-expressing MNPs within the adult human liver include a long-lived and/or self-renewing CD163^hi^CD206^hi^CX_3_CR1^hi^ subset, and can be supplemented by peripheral MNPs that undergo phenotypic reprogramming to partially resemble tissue-resident MNPs.

### The hepatic lymphoid compartment contains long-lived global and virus-specific T_RM_ that can be replenished from the blood

**Figure S3.**
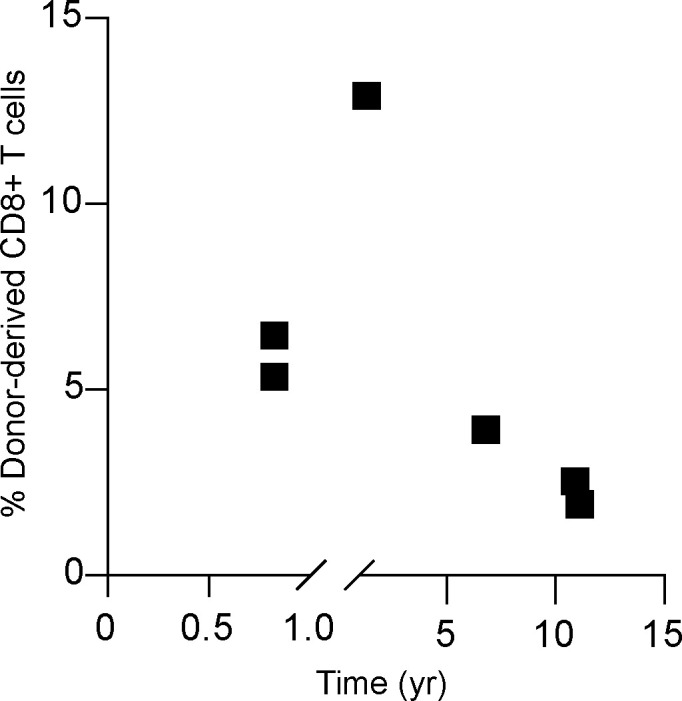
**Frequency of donor-derived CD8^+^ T cells by flow cytometry. **Frequency of donor-derived CD8^+^ T cells (gated on live, singlet, CD45^+^CD3^+^CD56^−^CD19^−^CD4^−^; *n* = 6; six independent experiments) identified in the allograft for each individual in relation to time of explant based on monoclonal antibody staining for donor-derived HLA-class I haplotype exemplified in Fig. S1 b.

**Figure 3.**
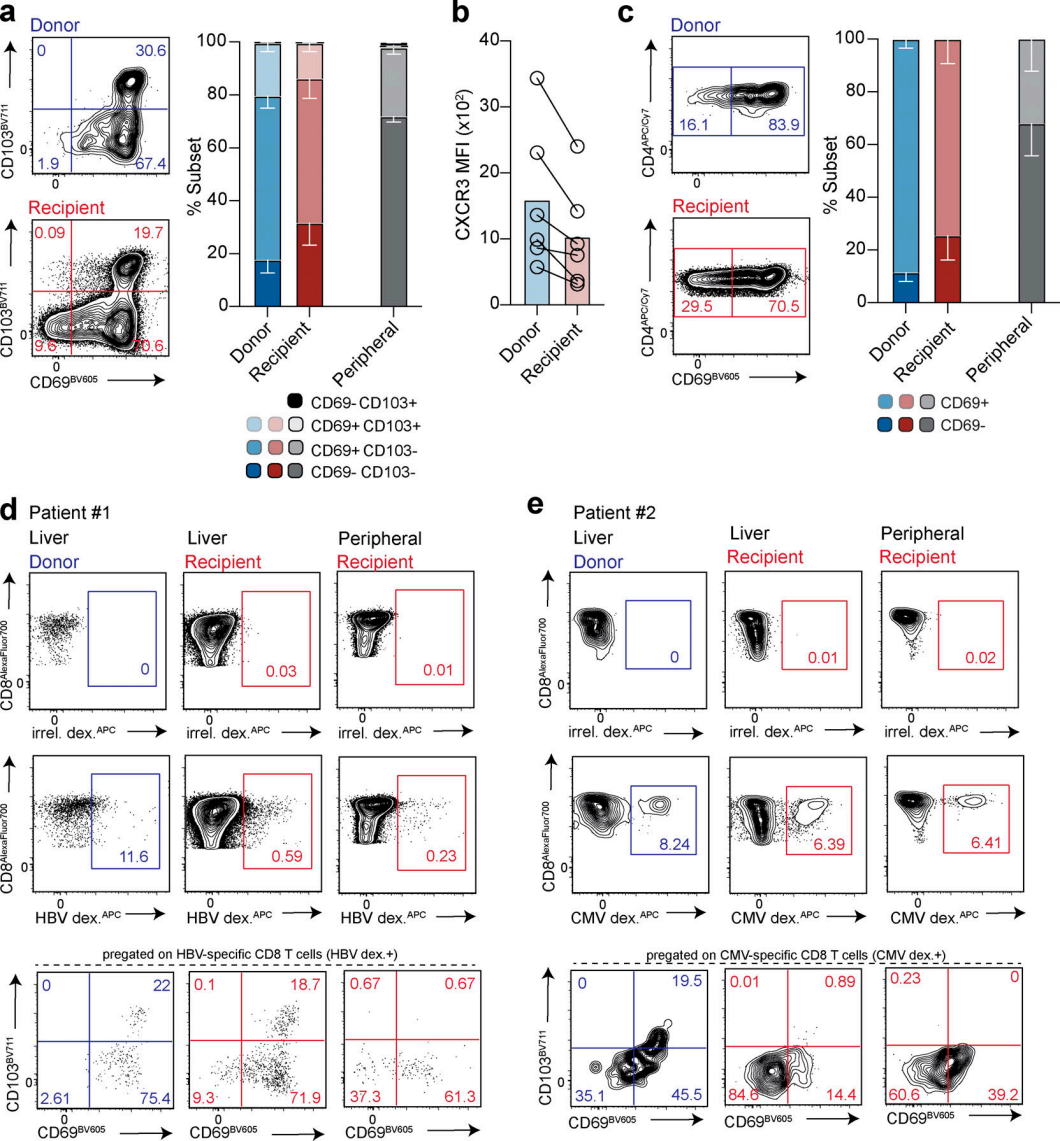
**Characterization of long-lived T_RM_ and their replenishment from the recipient-derived leukocyte pool. (a)** Summary data and representative flow-cytometric plots of CD69^+^CD103^+/−^ CD8^+^ T_RM_ within the donor-derived (blue) and recipient-derived (red) population (gated on live, singlet, CD45^+^CD3^+^CD56^−^CD19^−^CD4^−^; *n* = 6; six independent experiments) compared with peripheral blood taken at time of allograft removal (peripheral; *n* = 5; five independent experiments), using the gating strategy and HLA-class I antibody stain exemplified in Fig. S1, a and b. **(b)** CXCR3 expression (MFI; *n* = 6) on donor- and recipient-derived CD69^+^CD103^+^ CD8^+^ T_RM_ cells. **(c)** Summary data and representative flow-cytometric plots of CD4^+^ T_RM_ within the donor- and recipient-derived leukocytes (gated on live, singlet, CD45^+^CD3^+^CD56^−^CD19^−^CD8^−^; *n* = 6; six independent experiments) compared with peripheral blood taken at time of allograft removal (peripheral; *n* = 5; five independent experiments). **(d and e)** Identification and assessment of retention signals CD69 and CD103 on peripheral and intrahepatic CD8^+^ T cells specific for (d) HBV in a case of reactivated HBV infection within the allograft (further details in Table S1), or (e) CMV in a separate donor. Cells were stained with a panel of immunodominant epitope-based HLA-A*02-HBV or HLA-A*02-NLV peptide dextramers, and gated using an HLA-A*02 irrelevant peptide dextramer (irrel. dex.). Error bars, mean ± SEM.

We recently identified a population of CD8^+^ T cells in the human liver with phenotypic and functional features of CD8^+^ T_RM_ (Pallett et al., 2017). To test the longevity of human liver CD8^+^ T_RM_ we examined their persistence within the donor-derived pool of leukocytes using the same HLA-mismatched allografts characterized above. In all allografts examined, we were able to detect a persisting population of CD8^+^ T cells derived from the donor liver (2–6% of CD8^+^ T cells; Fig. S3 a). This donor-derived population tended to account for a lower proportion of the total intrahepatic CD8^+^ T cells the longer the duration of the allograft, but was still detectable in the two livers that had been transplanted 11 yr previously (Fig. S3 a), revealing for the first time the remarkable longevity of human liver-resident CD8^+^ T cell progeny in vivo. The majority of donor-derived CD8^+^ T cells persisting in the allograft were either CD69^+^CD103^+^ or CD69^+^CD103^−^ (Fig. 3 a), both of which are known to be characterized by core CD8^+^ T_RM_ transcriptional and functional signatures, although the latter are not completely excluded from the circulation (Kumar et al., 2017; Pallett et al., 2017). Such extreme longevity has also been demonstrated for liver-resident NK cells (Cuff et al., 2016), while human intestinal and lung allograft T_RM_ have been documented to survive at least 1 yr after transplantation (Bartolomé-Casado et al., 2019; Snyder et al., 2019).

We then looked within the large recipient-derived fraction to investigate whether T cells infiltrating from the circulation could acquire a tissue-resident phenotype and replenish intrahepatic CD8^+^ T_RM_. Recipient-derived CD8^+^ T cells infiltrating the liver were capable of acquiring high levels of CD69, and a proportion coexpressed CD103 (Fig. 3 a). These data suggest that T cells that circulate within the liver sinusoids can receive signals from the local microenvironment that up-regulate expression of the retention molecules CD69 and CD103, compared with recipient CD8^+^ T cells in the blood (Fig. 3 a). As expected, however, there tended to be a lower proportion of CD8^+^ T_RM_ in the recipient-derived pools than donor-derived pools. Moreover, the blood-derived CD8^+^ T_RM_ showed a less definitive residency profile, with lower levels of the liver-homing chemokine receptor CXCR3 than the donor-derived CD8^+^ T_RM_ (Fig. 3 b). CXCR3^hi^ CD8^+^ T_RM_ may have been preferentially enriched among the small persisting donor pool by being better equipped for retention following the pretransplant perfusion procedure (which removes large numbers of CD8^+^ T_RM_; Pallett et al., 2017), and/or equipped to receive additional signals for long-term survival. Allograft studies in the intestine and lung have likewise found that circulating T cells replenishing the resident pool have incomplete acquisition of the residency program (Bartolomé-Casado et al., 2019; Snyder et al., 2019).

A small population of CD4^+^ T cells persisting from the donor leukocyte pool was also detectable in all liver allografts. The majority of this long-lived population expressed high levels of CD69 (Fig. 3 c), with minimal CD103 expression, in line with the phenotype of CD4^+^ T_RM_ in other human tissues (Turner and Farber, 2014). As with CD8^+^ T cells, a fraction of the peripheral CD4^+^ T cells infiltrating the liver allograft up-regulated CD69, suggestive of acquisition of tissue residency (Fig. 3 c).

To study the potential for virus-specific T cells to acquire long-term liver residence and/or be supplemented by peripheral responses, we took advantage of access to an HLA-mismatched liver from a donor with chronic hepatitis B virus (HBV) infection (reactivated following immunosuppression and only partially suppressed with antivirals). A population able to bind a panel of HLA-A2/HBV peptide multimers (and not binding an irrelevant peptide-loaded HLA-A2 multimer) was detectable among the small fraction of CD8^+^ T cells of donor (HLA-A2^+^) origin compartmentalized in the liver 11 yr after transplantation (Fig. 3 d). In addition, a small percentage of the recipient-derived (HLA-A2^−^) CD8^+^ T cells in both the blood and liver were able to bind the HLA-A2/HBV peptide multimer panel (Fig. 3 d). Donor, and to a slightly lesser extent, recipient intrahepatic (but not peripheral) HBV multimer-binding cells displayed a CD8^+^ T_RM_ phenotype (Fig. 3 d), compatible with local antigen recognition (Kim et al., 2020). Donor and recipient-derived CD8^+^ T cells directed against a CMV epitope presented by the donor HLA were also identified in another HLA-mismatched allograft; recipient-derived infiltrating CMV-specific CD8^+^ T cells did not acquire a T_RM_ phenotype, suggesting a lack of ongoing cognate antigen recognition within the liver (Fig. 3 e). Although preliminary, these findings suggest long-term persistence of donor-derived virus-specific CD8^+^ T_RM_ and potential supplementation by recipient responses. The T cells we detected binding multimers covering viral epitopes restricted by allogeneic HLA could represent responses primed within the infected allograft (Rosen et al., 2004) or the commonly recognized cross-reactivity between allograft-specific and virus-specific memory T cells (Amir et al., 2010).

Taken together, these data show that tissue immunity can be sustained by the progeny of, and/or long-lived, local intrahepatic populations of CD4^+^ and CD8^+^ T_RM_, supplemented by newly recruited T cells from the blood that acquire at least some of the characteristics of T_RM_. The longevity of human T_RM_ supports their potential therapeutic utility in providing sustained immunosurveillance of residual virus (e.g., in the functional cure of HBV) or tumor.

### Sampling hepatic vasculature and lymph nodes to test for egress of hepatic CD8^+^ T_RM_

**Figure 4.**
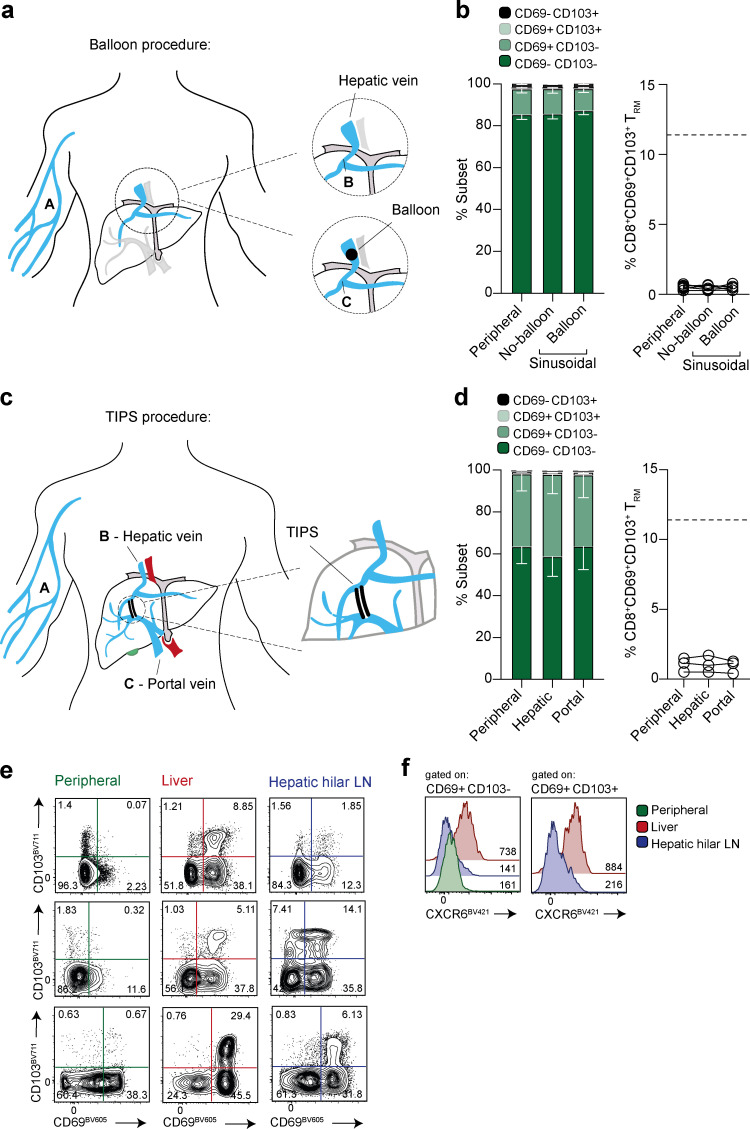
**Assessment of egress of CD8^+^ T_RM_ in vasculature and draining lymph nodes. (a)** Schematic showing experimental sampling of peripheral blood (A) compared with liver sinusoidal blood taken following hepatic vein catheterization, before (B) and during (C) balloon inflation to temporarily occlude the vein. **(b)** Summary data of CD69^+^ CD103^+^ CD8^+^ T_RM_ frequencies (gated on live, singlet, CD45^+^CD3^+^CD56^−^CD19^−^CD4^−^; *n* = 13; 13 independent experiments) matched peripheral blood and liver sinusoidal blood ± balloon occlusion. **(c)** Schematic showing experimental sampling of peripheral (A), hepatic (B) and portal venous (C) blood of individuals undergoing a TIPS procedure. **(d)** Summary data of CD69^+^CD103^+^ CD8^+^ T_RM_ in matched peripheral, hepatic, and portal vein samples (*n* = 3; three independent experiments). **(e)** CD69^+^CD103^+/−^ CD8^+^ T_RM_ in matched peripheral blood, resected liver tissue and hepatic hilar lymph nodes (*n* = 3; three independent experiments). **(f)** CXCR6 expression (MFI) on CD69^+^CD103^+^ CD8^+^ T_RM_ or CD69^+^CD103^−^ CD8^+^ T_RM_ isolated from liver or hepatic hilar lymph node (compared with peripheral blood, where possible; *n* = 3; three independent experiments). Dotted line on figures represents the mean frequency of CD8^+^ T_RM_ within control liver tissue (as previously published in Pallett et al., 2017). Error bars, mean ± SEM.

The inability to detect CD69^+^CD103^+^CD8^+^ T cells when sampling blood taken from peripheral veins (Pallett et al., 2017) is highly suggestive that they are unable to egress, representing a key characteristic of tissue residency. However, it is difficult to definitively conclude from this that they are completely compartmentalized in the human liver. CD8^+^ T_RM_ leaving the liver vasculature at low frequency would be difficult to detect following dilution in the peripheral circulation. We therefore made use of unusual access to the hepatic vasculature, allowing direct sampling of hepatic venous blood, to test for low-level egress of CD8^+^ T_RM_ (Fig. 4 a). Samples taken from 13 patients with liver cirrhosis undergoing hepatic vein catheterization failed to detect any CD69^+^CD103^+^CD8^+^ T_RM_. Inflation of a balloon to temporarily obstruct the hepatic venous outflow permitted concentrated sampling of the accumulated cellular contents effluxing hepatic sinusoids. This procedure again failed to reveal any low-level egress of CD69^+^CD103^+^CD8^+^ T_RM_ from the hepatic sinusoids (with frequencies of <1%, equivalent to the matched peripheral blood; Fig. 4 b).

The liver is the only organ in the body that receives blood from a dual source; in addition to the hepatic artery supply, a large proportion of its blood is delivered by the portal vein, draining directly from the intestinal and splenic veins. Leukocytes coming into the liver via the portal vein could therefore have transited through the gut first, which is also known to contain a large population of CD69^+^CD103^+^CD8^+^ T_RM_ (Bartolomé-Casado et al., 2019; Szabo et al., 2019). Following hepatic vein sampling, the transjugular intrahepatic portosystemic shunt (TIPS) procedure (performed in patients with liver cirrhosis) then allowed access to blood samples from the portal vein (schematic, Fig. 4 c). Portal and hepatic venous sampling again gave the same phenotypic profile as matched peripheral blood, with no detectable CD8^+^ T_RM_ egressing from the gut or liver (Fig. 4 d). These data therefore provide further support for the tissue sequestration of human hepatic and intestinal CD8^+^ T_RM_, suggesting they are unable to egress into the draining vasculature.

Although hepatic CD8^+^ T cells can reside within the sinusoidal vasculature (Fernandez-Ruiz et al., 2016), if some transmigrate through the hepatic endothelium, they would be expected to exit the liver via the lymphatic drainage into local lymph nodes, rather than directly into the hepatic vein. We therefore examined hepatic hilar (liver-draining) lymph nodes for evidence of CD8^+^ T_RM_. In three cases we were able to simultaneously extract leukocytes from blood, liver tissue, and hepatic hilar lymph nodes for flow-cytometric assessment (Fig. 4 e). In all cases, CD69^+^CD103^+^CD8^+^ T_RM_ were detectable in matched liver and these major liver-draining lymph nodes (2–14% of CD8^+^ T cells), but not in blood. The detection of CD8^+^ T_RM_ in a liver-draining lymph node is in line with recent murine data showing the capacity for emigrants from nonlymphoid tissues to selectively acquire residence in the local organ-draining lymph node (Beura et al., 2018). However the CD8^+^ T_RM_ within the hepatic hilar lymph nodes lacked the expression of CXCR6 seen on those within the liver (Fig. 4 f); this suggested that either CD8^+^ T_RM_ capable of migrating from the liver to the draining lymph node down-regulate CXCR6, or those within the lymph node represented an independent population that had developed residence in situ, in line with the identification of CD8^+^ T cells with a tissue-resident signature in many human lymphoid sites (Buggert et al., 2018; Kumar et al., 2017; Miron et al., 2018). Future studies could use TCR clonotype tracking of donor allograft T cells in draining lymph nodes to distinguish these two scenarios.

In summary, we have used HLA discordant donor-recipient samples to probe the turnover of the liver myeloid and lymphoid compartments in parallel. Within this small clinically heterogeneous cohort, we were able to characterize some consistent features of the intrahepatic donor and recipient immune landscape. The vast majority of liver leukocytes were rapidly replenished from the blood, with recapitulation of the characteristic composition of the liver myeloid and lymphoid pools. However, despite the setting of disrupted homeostasis resulting from transplantation, we were able to detect small long-lived populations of liver MNPs and T cells. Subsets of MNP (CX_3_CR1^hi^/CD163^hi^/CD206^hi^CD68^+^) and T cells (CXCR3^hi^CD69^+^CD103^+/−^), including antigen-specific T cell responses, had the capacity for longevity/self-renewal for more than a decade in the human liver. Incoming peripheral MNPs and T cells could acquire some features of the persistent donor-derived populations (CD68/CD206 and/or CD69±CD103, respectively) to replenish the resident pools, and T_RM_ were detectable in the local draining lymph nodes but not egressing into the hepatic vasculature. Our findings on the dynamics of the human liver immune landscape, confirming prototypic residency features of key frontline immune sentinels (KCs and T cells), have direct implications for understanding graft tolerance and advancing hepatic immunotherapy (Beura et al., 2017).

## Materials and methods

### Ethical approval

This study complies with the declaration of Helsinki and was approved by local ethics boards: (1) UK: National Health Service Research Ethics Committee (REC) for the Royal Free Hospital (RFH); and (2) Spain: National Board Comite Etico De Investigacion Clinica for Hospital Clínic de Barcelona.

All individuals recruited gave written informed consent before inclusion in the study. Tissue and peripheral blood samples were obtained through the Tissue Access for Patient Benefit scheme at the RFH, approved by the University College London (UCL) RFH BioBank Ethical Review Committee (UCL/RFH Biobank; REC reference 11/WA/0077) and included samples from individuals undergoing retransplantation (i.e., receiving a second liver transplant) for disease recurrence where the primary donor organ was HLA-mismatched; control nontransplanted liver tissue and paired peripheral blood from individuals undergoing liver resection surgeries for colorectal metastatic liver disease; and hepatic hilar lymph nodes (surplus to diagnostic requirements) removed at the time of organ transplantation. Blood samples obtained during hepatic vein catherization, with and without additional balloon inflation to occlude the vein, were obtained from the Hospital Clínic de Barcelona (approval reference HCB/2017/0806). Further venous blood samples were obtained from a peripheral vein, the hepatic vein and the portal vein from individuals undergoing the TIPS procedure at the RFH, approved by UCL/RFH Biobank (REC reference 16/WA/0289).

### Sample collection

Explanted liver tissue samples obtained from patients undergoing retransplantation (i.e., receiving a second liver transplant) were examined in the study where there was an HLA class I mismatch between the initial liver donor and recipient, as determined by HLA-haplotyping PCR by A. Nolan (National Health Service, London, UK) or MRC Weatherall Institute of Molecular Medicine Sequencing Facility (Oxford, UK). All transplant recipients received a standard immunosuppressive regimen of FK506 (Prograf). Matched peripheral blood samples were collected at the time of retransplantation for peripheral blood mononuclear cell (PBMC) isolation. Full details of transplant recipients and donors including disease pathologies and treatment regimen are included in Table S1.

For comparison, nontransplanted liver tissue samples distal to the tumor site were also obtained from individuals undergoing surgery for a number of indications, including colorectal metastatic liver disease and hepatocellular carcinoma (HCC; referred to as liver).

13 blood samples were obtained from the hepatic veins of individuals with liver cirrhosis undergoing hepatic vein catheterization (for transjugular liver biopsy or hepatic venous pressure gradient measurement). Blood samples leaving the sinusoids were collected from the hepatic vein with and without additional balloon inflation to temporarily occlude the vein, permitting measurement of leukocyte accumulation (see cartoon in Fig. 4 a).

Further blood samples were taken from three individuals who had undergone a TIPS procedure, providing access to the portal and hepatic veins, alongside a matched peripheral blood sample (see cartoon in Fig. 4 c).

### PBMC/intrahepatic leukocyte (IHL)/lymph node sample processing

PBMC were isolated from heparinized blood by density centrifugation using Pancoll (Pan Biotech) and used immediately for flow-cytometric analysis.

Resected/explanted livers were processed to isolate IHLs. Liver samples were dissected into smaller pieces and enzymatically digested in 0.01% collagenase IV (Thermo Fisher Scientific) and 0.001% DNase I (Sigma-Aldrich). A GentleMACs (Miltenyi Biotech) was used to further mechanically digest the liver material, which was then filtered through 70-µM cell strainers to remove debris. The resulting single-cell suspension underwent centrifugation on a 30% Percoll (GE Healthcare) gradient to remove parenchymal cells. IHLs were then isolated by density centrifugation using Pancoll.

Leukocytes from hepatic hilar lymph nodes were obtained by dissecting the tissue into smaller pieces and filtering through 70-µM cell strainers to remove debris. The resulting single-cell suspension underwent centrifugation on a Pancoll gradient, as before.

In all cases, samples not used immediately were frozen in 10% DMSO (Sigma-Aldrich) in FBS (Sigma-Aldrich) and stored in accordance with the Human Tissue Act.

### Flow cytometry

Multi-parametric flow cytometry was used for the phenotypic and functional analysis of PBMCs/IHLs/lymph node leukocytes. Cells were stained with saturating concentrations of surface monoclonal antibodies diluted in 50%-Brilliant Violet Buffer (BD Bioscience):50%-1× PBS (Thermo Fisher Scientific). Dead cells were excluded from analysis using a fixable viability dye (Thermo Fisher Scientific). Following surface staining, cells were fixed (and permeabilized) with Cytofix/Cytoperm (BD Bioscience).

Where necessary, intracellular proteins were detected using saturating concentrations of monoclonal antibodies in a 0.1% saponin-based buffer (Sigma-Aldrich). All samples were acquired on a BD Bioscience Fortessa-X20 and analyzed using FlowJo v.9 (TreeStar/BD Bioscience). Full details of all monoclonal antibodies used for flow-cytometric analysis are given in Table S2.

### Dextramer staining for the identification of virus-specific T cells

The frequency and phenotype of HBV-specific T cells was analyzed using HLA-A*02-restricted HBV dextramers (Immudex) against the following specificities: core 18–27 (FLPSDFFPFV), envelope 183–191 (FLLTRILTI), envelope 335–342 (WLSLLVPFV), envelope 348–357 (GLSPTVWLSV), polymerase 455–463 (GLSRYVARL), and polymerase 502–510 (KLHLYSHPI). For CMV-specific T cells, the HLA-A*02-restricted NLVPMVATV peptide dextramer (Immudex) was used. Cells were stained with dextramers at 37°C in 1× PBS, washed twice in RPMI-1640, and left to rest for 1 h before further staining. Samples were then stained with phenotypic markers, including HLA class I antibodies, as above. Dextramers loaded with an irrelevant peptide were used in parallel to control for nonspecific binding. Dead cells, doublets, and CD19^+^ B cells were removed during analysis to minimize nonspecific binding contamination.

### Online supplemental material

Fig. S1 provides representative gating strategies used to identify donor- and recipient-derived CD45^+^ leukocytes, MNPs, and DC subsets, based on HLA-class I monoclonal antibody staining. The frequency of donor-derived CD45^+^ leukocytes identified in the allograft in relation to time to explant is also plotted (Fig. S1 c). Fig. S2 contains data characterizing the coexpression of phenotypic markers on MNP profiled in Fig. 3, and the expression of HMOX1, on long-lived donor-derived and infiltrating recipient-derived MNP. Fig. S3 shows the frequency of donor-derived CD8^+^ T cells identified in the allograft, plotted in relation to time to explant. Table S1 provides clinical characterization of the patients undergoing liver retransplantation included in this study. Table S2 lists details of the monoclonal antibodies used.

## Supplementary Material

Table S1 shows clinical details of patients undergoing re-transplantation where there was an HLA-class I mismatch between the initial liver donor and recipient.

Table S2 shows details of monoclonal antibodies used for flow-cytometric analysis.
