# Supplementary material for: Longevity and replenishment of human liver-resident memory T cells and mononuclear phagocytes
Source: J Exp Med. 2020 Jun 30;217(9):e20200050. doi: 10.1084/jem.20200050 (PMC7478732; doi:10.1084/jem.20200050)
Supplement: Table S1 — shows clinical details of patients undergoing re-transplantation where there was an HLA-class I mismatch between the initial liver donor and recipient. [file JEM_20200050_TableS1.docx]

Table S1. Clinical details of patients undergoing re-transplantation where there was an HLA-class I mismatch between the initial liver donor and recipient

| Recipient | | | Donor | | | Original transplant date and time to re-transplantation | Clinical details including primary disease and indication for re-transplantation |
| --- | --- | --- | --- | --- | --- | --- | --- |
| HLA | Gender | Virology | HLA | Gender | Virology |  |  |
| A03  A24  B51  B07 | M | Anti-HBsAb^−^ pre-transplant | A02  A31  B60  B51 | M | Anti-HBcAb^+^ anti-HBsAg^+^ | 2007  11 yr 1 mo | Primary disease: PSC |
|  |  |  |  |  |  |  | Donor organ: HBV resolved infection, reactivation in recipient treated with lamivudine, then tenofovir |
|  |  |  |  |  |  |  | Reason for re-transplantation: PSC recurrence with cirrhosis |
| A02:01  A24:02  B39:06  B44:02 | M | CMV IgG^+^ | A03  A29  B14:02  B44 | M | (Unknown) | 2018  8 mo | Primary disease: PSC |
|  |  |  |  |  |  |  | Reason for re-transplantation: with bile leak/ hepatic artery pseudoaneurysm |
|  |  |  |  |  |  |  | No graft cirrhosis or chronic cellular rejection |
| A01:01  A30:01  B08:01  B13:02 | M | HCV^+^ anti-HBcAb^−^ HBsAg^−^ | A01:01 A02:01 B08:01 B18:01 |  | CMV IgG^+^HBsAg^−^ anti-HBcAb^−^HIV IgG^−^HCV IgG^−^ | 2018  8 mo | Primary disease: HCV-related HCC treated with Peg-IFN and sofosbuvir/ribavirin, TACE for HCC 2017 |
|  |  |  |  |  |  |  | Reason for re-transplantation: chronic graft dysfunction due to ischemic cholangiopathy |
|  |  |  |  |  |  |  | No graft cirrhosis |
|  |  |  |  |  |  |  | Early stage chronic rejection |
| A01  A03  B07  B47 | F | HBsAg^−^anti-HBcAb^+^HCV IgG^−^CMV IgG^+^EBV IgG^+^ | A02  A03  B07 | M | (Unknown) | 2008  11 yr | Primary disease: drug-induced liver failure |
|  |  |  |  |  |  |  | Reason for re-transplantation: biliary complications with allograft cirrhosis |
|  |  |  |  |  |  |  | Early-stage antibody-mediated chronic rejection |
| A01:01  A31:01  B40:01  B40:01 | M | anti-HCV Ab^+^HCV RNA^−^(genotype3) HBsAg^−^anti-HBcAb^+^ anti-HBsAb^+^CMV IgG^+^EBV IgG^+^HIV IgG^−^ | A02  A03  B08 | F | Anti-HBcAb^-^HBsAg- anti-HCVAb^−^CMV IgG^−^HIV IgG^−^ | 2012  6 yr 8 mo | Primary disease: ALD/HCV-related HCC treated treated with ribavirin and peg-IFNa |
|  |  |  |  |  |  |  | Reason for re-transplantation: hepatic artery thrombosis (with multiple biliary abscesses) |
|  |  |  |  |  |  |  | No evidence of allograft cirrhosis or cellular rejection |
| (Unknown) A09 antibody negative A02 antibody pos |  |  | A09  A24  B18  B35 |  | Anti-HBcAb^−^HBsAg^−^anti-HCVAb^−^CMV IgG^-^HIV IgG^−^ | 2010  6 mo | Primary disease: HCV-related HCC |
|  |  |  |  |  |  |  | Reason for re-transplantation: cholangiopathy |

ALD, alcoholic liver disease; F, female; HCV, hepatitis C virus; M, male; pos, positive; PSC, primary sclerosing cholangitis; TACE, transarterial chemoembolization.
