# Supplementary material for: Longevity and replenishment of human liver-resident memory T cells and mononuclear phagocytes
Source: J Exp Med. 2020 Jun 30;217(9):e20200050. doi: 10.1084/jem.20200050 (PMC7478732; doi:10.1084/jem.20200050)
Supplement: Table S2 — shows details of monoclonal antibodies used for flow-cytometric analysis. [file JEM_20200050_TableS2.docx]

Table S2. Details of monoclonal antibodies used for flow-cytometric analysis

| Antigen | Fluorochrome | Clone | Supplier | Dilution |
| --- | --- | --- | --- | --- |
| **Lymphoid panel** | | | | |
| CD45 | BUV805 | HI30 | BD Bioscience (564915) | 1:100 |
| CD3 | BUV395 | SP34-2 | BD Bioscience (564117) | 1:100 |
| CD8 | Alexa Fluor 700 | RPA-T8 | BD Bioscience (561453) | 1:100 |
| CD4 | APC-Cy7 | RPA-T4 | BD Bioscience (566319) | 0.5:100 |
| CD56 | PE/Dazzle-594 | NCAM-HCD56 | Biolegend (318348) | 0.5:100 |
| CD19 | BV786 | HIB19 | BD Bioscience (740968) | 1.5:100 |
| CD69 | BV605 | FN50 | Biolegend (310938) | 0.5:100 |
| CD103 | BV711 | Ber-ACT8 | Biolegend (350222) | 2:100 |
| CXCR3 (CD186) | PerCP-Cy5.5 or BB700 | 1C6/CXCR3 | BD Bioscience (560832/566533) | 3:100 |
| HLA-DR | BV510 | G46-6 | BD Bioscience (563083) | 1:100 |
| PD-1 | PE | EH12.2H7 | Biolegend (329906) | 2:100 |
| CD3 | PE/Dazzle-594 | UCHT1 | Biolegend (300335) | 0.5:100 |
| CD20 | PE/Dazzle-594 | 2H7 | Biolegend (302348) | 1:100 |
| CD19 | PE/Dazzle-594 | HIB19 | Biolegend (302252) | 1:100 |
| **Myeloid panel** | | | | |
| CD14 | APC/Cy7 | M5E2 | Biolegend (301820) | 2:100 |
| CD14 | BV510 | M5E2 | BD Bioscience (740163) | 1:100 |
| CD16 | Alexa Fluor 700 | 3G8 | Biolegend (302026) | 1:100 |
| CD16 | BV711 | 3G8 | Biolegend (302043) | 1:100 |
| CX_3_CR1 | PerCP/Cy5.5 | 2A9-1 | Biolegend (341613) | 1:100 |
| CCR2 (CD192) | BV605 | LS132.1D9 | BD Bioscience (747850) | 1:100 |
| CD206 | BV421 | 15-2 | Biolegend (321125) | 2:100 |
| HMOX1 | Unconjugated | HO-1-1 | Thermo Fisher Scientific (MA1-112) | 1:100 |
| CD68^a^ | APC | Y1/82A | Biolegend (333809) | 3:100 |
| CD163 | BV785 | GHI/61 | Biolegend (333632) | 2:100 |
| CD123 | BUV395 | 7G3 | BD Bioscience (564195) | 2:100 |
| CD11c | BV421 | B-ly6 | BD Bioscience (562561) | 2:100 |
| **HLA-haplotyping** | | | | |
| HLA-A2 | FITC | BB7.2 | BioRad (MCA2090) | 3:100 |
| HLA-A2 | PE/Cy7 | BB7.2 | Biolegend (343314) | 3:100 |
| HLA-A3 | PE | GAP.A3 | Thermo Fisher Scientific (12-5754-42) | 3:100 |
| HLA-A9 | APC | Rea127 | Miltenyi Biotech (130-099-539) | 3:100 |

^a^Intracellular stain.
